# Association between Low-Dose Computed Tomography Results and 1-Year Smoking Cessation in a Residential Smoking Cessation Program

**DOI:** 10.3390/ijerph19095510

**Published:** 2022-05-01

**Authors:** Da-Som Shin, Hye-Mi Noh, Hong Ji Song, Kyung Hee Park, Young-Gyun Seo, Yu-Jin Paek

**Affiliations:** Department of Family Medicine, Hallym University Sacred Heart Hospital, College of Medicine, Hallym University, Anyang 14068, Korea; ektha0602@hallym.or.kr (D.-S.S.); hongji@hallym.or.kr (H.J.S.); beloved@hallym.or.kr (K.H.P.); yg035@hallym.or.kr (Y.-G.S.)

**Keywords:** COVID-19, lung cancer, smoking cessation, tobacco, computed tomography

## Abstract

The COVID-19 pandemic is a global health threat. Smoking and smoking-related lung diseases are risk factors for severe COVID-19 infection. This study investigated whether low-dose computed tomography (LDCT) scan results affected the success of 1-year smoking cessation. The Gyeonggi Southern Smoking Support Center performed the residential smoking cessation program from January to December 2018. During the program, LDCT was performed on 292 participants; 6 months later, follow-up via telephone or visit was conducted. Among the 179 participants who succeeded in smoking cessation for 6 months, telephone follow-up was conducted to determine whether there was a 12-month continuous smoking cessation. In order to evaluate the association between LDCT results and 12-month continuous abstinence rate (CAR), logistic regression was used to estimate the odds ratio (OR) and 95% confidence interval (CI). The CARs at 6 and 12 months were 61.3% and 31.5%, respectively. Indeterminate or suspicious malignant lung nodules were associated with a higher 12-month CAR (OR, 3.02; 95% CI, 1.15–7.98), whereas psychiatric history was associated with a lower 12-month CAR (OR, 0.06; 95% CI, 0.03–0.15). These results suggest that abnormal lung screening results may encourage smokers to quit smoking.

## 1. Introduction

The COVID-19 pandemic is ongoing worldwide. Although there are inconsistent results on whether smoking increases the risk of COVID-19 infection [1,2], smoking is a risk factor for severe COVID-19 infection and death [3]. Particularly, patients with smoking-related lung diseases such as chronic pulmonary obstructive disease and lung cancer are more vulnerable to COVID-19 [4]. The prevalence of smoking among Korean adults decreased to 20.6% in 2020 from 35.1% in 1998 [5]. However, during the pandemic, the Ministry of Health and Welfare of South Korea stopped public health centers that provided national smoking cessation services such as face-to-face counseling to prevent the spreading of COVID-19. The sales of conventional cigarettes increased from 3063 in 2019 to 3209 million packs in 2020. Furthermore, the number of smokers participating in national smoking cessation services rapidly decreased during the pandemic to 89,283 in the first half of 2020 from 358,966 in 2019 [6].

Lung cancer is the leading cause of cancer-related deaths, accounting for approximately 1.8 million deaths worldwide [7]. The incidence rate of lung cancer in Korea ranked third in the overall cancer incidence rates in 2018. For older adults aged 65 years and above, the incidence rate of lung cancer is higher than that of younger adults, ranking first in men and second in women [8]. In addition, the number of deaths from lung cancer has continuously increased from 13,246 in 2004 to 17,980 in 2017 among Koreans [9].

Lung cancer screening is advantageous for the early detection and treatment of lung cancer. In the National Lung Screening Test (NLST) conducted in the United States, screening tests were conducted on high-risk lung cancer groups (aged 55–74 years) with a history of smoking for more than 30 pack-years. A comparison between the group examined with low-dose computed tomography (LDCT) and the control group examined with simple chest radiography showed 20% and 7% reductions in lung cancer mortality and all-cause mortality in the LDCT group, respectively [10]. In the Netherlands and Belgium, the Nederlands Leuvens Screening ONderzoek (NELSON) trial showed that lung cancer mortality rates decreased by 24% for men and 33% for women in participants (aged 50–74 years) who underwent volume CT screening compared to those without screening [11].

Smoking cessation is the most effective strategy for preventing lung cancer-related deaths [12]. Beyond the potential benefits of early detection of lung cancer in CT screening, smoking cessation counseling has been considered an essential factor in lung cancer screening programs in the United States [13]. Abnormal results of lung cancer screening tests, such as suspicious malignancy or other pulmonary diseases, can be used to encourage smoking cessation as a “teachable moment”. To date, results regarding the effect of CT screening results on smoking cessation have been inconsistent. Several studies have reported that participants with abnormal findings on LDCT had a higher success rate of smoking cessation than those with normal LDCT [14,15,16]. However, some studies have shown no difference in the prolonged abstinence rate between the group with normal findings and the group with abnormal findings on LDCT [17,18].

In South Korea, the national lung cancer screening has been conducted since 2019. However, no Korean study has evaluated the association between lung cancer screening results and smoking cessation. In addition, since 2015, the Korean government has funded residential smoking cessation programs that are held for 5 days for participants with a smoking duration ≥20 years in 18 regional tobacco control centers. As one of the regional tobacco control centers, we operate smoking cessation programs and evaluate lung screening tests by LDCT for participants. In our previous study, we identified factors associated with 6-month smoking cessation [19].

This study aimed to (1) investigate the rate of 12-month continuous smoking abstinence in a residential smoking cessation program and (2) estimate whether the results of LDCT scans affect the success of smoking cessation.

## 2. Materials and Methods

### 2.1. Study Participants

This study included participants who had completed a 5-day smoking cessation camp program conducted by the Gyeonggi Southern Smoking Support Center between January 2018 and December 2018. According to the National Tobacco Control Center of the Ministry of Health and Welfare of Korea, the criteria for selecting participants were as follows: (1) participants with a smoking history of 20 years or more who have attempted smoking cessation more than twice and/or (2) those with chronic diseases (e.g., dyslipidemia, diabetes mellitus, and hypertension). Of the 313 participants in the smoking cessation camp 2018, we included 292 participants who underwent LDCT as the study population. Of 292 participants, 179 were successful in quitting smoking for 6 months. For the 179 participants who succeeded in smoking cessation for 6 months, telephone counseling was conducted to confirm whether smoking cessation continued for 1 year. All participants provided written informed consent prior to participating in this study. The study protocol was approved by the Institutional Review Board of Hallym University Sacred Heart Hospital (approval number: 2019-01-023).

### 2.2. Measurements

The demographic and socioeconomic characteristics of the participants were collected. Data on age, sex, education, alcohol consumption, exercise, chronic comorbidities (hypertension, diabetes mellitus, dyslipidemia, coronary artery disease, cerebrovascular disease, cancer, and psychiatric disorders), information about smoking (smoking duration, cigarettes per day, results of the Fagerström Test for Nicotine Dependence [FTND]), and smoking cessation medications were collected. For lung cancer screening, LDCT was evaluated in all participants. LDCT was performed by an experienced radiologist. Pulmonary nodules were divided into four categories: (1) normal, (2) benign nodule, (3) indeterminate nodule requiring follow-up scan, and (4) suspicious malignancy. In addition, abnormal findings, such as emphysema, interstitial lung disease, extrapulmonary neoplasm, and coronary calcification, were noted in the CT reports.

### 2.3. Intervention and Follow-Up

As mentioned in the previous study, a multidisciplinary team (physicians, nurses, and counselors) of the regional tobacco control center provided the 5-day residential smoking cessation program. Physicians prescribed smoking cessation medications (e.g., varenicline, bupropion, or nicotine replacement therapy [NRT]) to participants. The program consisted of lectures on smoking cessation education, intensive psychological counseling, and counseling on the results of LDCT screening [19]. Telephone follow-up or follow-up visit at 2, 4, 6, 12, 18, and 24 weeks after the residential smoking cessation program was conducted. Continuous abstinence was evaluated biochemically via urine cotinine (COT) test, exhaled-air carbon monoxide (CO) level test (visit), or self-reports (telephone). The cut-off value for urine COT was 20 ng/mL (COT urine rapid test), and the cut-off value for exhaled CO was 5 ppm. Non-responders for >2 months were considered to have a smoking relapse.

**Figure 1:**
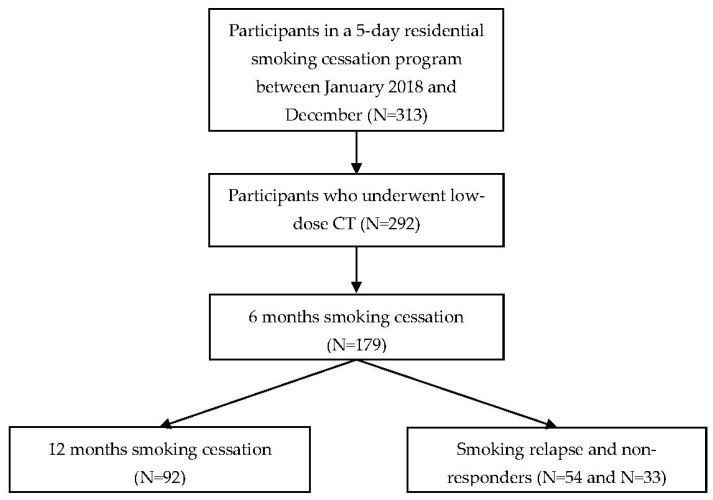
Flowchart of study subjects.

Among the 179 participants who succeeded in smoking cessation for 6 months, follow-up was conducted only by telephone interview to determine whether there was 12-month continuous smoking cessation (continuous abstinence rate [CAR]) by asking the following question: “After participating in the smoking cessation camp, have you maintained smoking cessation continuously for 12 months?”. In total, 92 participants reported that they continuously succeeded in smoking cessation for 12 months, 54 failed, and 33 did not respond to telephone counseling. We considered non-respondents to be failures (Figure 1).

### 2.4. Statistical Analysis

Continuous variables were compared using a t-test, and categorical variables were compared using Fisher’s exact test and the chi-squared test. Multivariate logistic regression analysis was used to obtain the odds ratio (OR) and 95% confidence interval (CI) to investigate the predictors of 6- and 12-months smoking cessation. Variables that were significantly different between ex-smoker and smoking relapse groups were included in the models. We adjusted for age and sex in Model 1, and Model 2 included all significant univariable predictors. For all tests, the Statistical Package for the Social Sciences version 23.0 (IBM Co., Armonk, NY, USA) was used. Statistical significance was set at a *p*-value < 0.05.

## 3. Results

Table 1 shows the characteristics of the study participants according to 6- and 12-month smoking cessation. The success rates at 6 and 12 months were 61.3% and 31.5%, respectively. A comparison of success rates by sex showed that both the 6-month CARs (62.6% vs. 45.5%) and the 12-month CARs (33% vs. 13.6%) were higher in men than in women. Those who quit for 6 months had a larger proportion of higher education, regular exercise, history of dyslipidemia and cerebrovascular disease, absence of psychiatric disease, lower smoking pack-years, and FTND score. Those who quit for 12 months had a higher proportion of history of cerebrovascular disease and absence of psychiatric disease (Table 1).

Table 2 shows the results of the LDCT screening of the study participants. Of 30 participants who had indeterminate or suspicious nodules, 29 participants (96.7%) had indeterminate nodules, and 1 participant (3.3%) had a suspicious nodule. The 6-month CARs and the 12-month CARs were the participants with normal (59.6% and 27.6%), benign nodules (55.7% and 25.5%), and indeterminate or suspicious malignancy nodules (90% and 73.3%). Both 6- and 12-month ex-smokers had a higher proportion of indeterminate or suspicious malignant lung nodules on LDCT than participants who failed smoking cessation (*p* = 0.001 and *p* < 0.001). Moreover, 6-month ex-smokers had a higher proportion of interstitial lung disease and coronary artery calcification in LDCT than participants who failed smoking cessation (*p* = 0.046 and *p* = 0.026). Furthermore, 12-month ex-smokers had a lower proportion of emphysema on LDCT than participants who failed smoking cessation, despite being statistically insignificant (*p* = 0.057).

Table 3 shows the multivariate logistic regression analysis of predictors for 6-month smoking cessation. Higher educational level (OR, 4.37; 95% CI, 1.36–14.08) and performing regular exercise (OR, 2.41; 95% CI, 1.22–4.76) were associated with a higher 6-month CAR. Having a psychiatric disease (OR, 0.06; 95% CI, 0.03–0.12) was associated with a lower 6-month CAR. A higher FTND score was associated with a lower 6-month CAR in Model 1 (OR 0.88; 95% CI, 0.80–0.98); however, it lost statistical significance in Model 2 (OR 0.88; 95% CI 0.77–1.00).

Table 4 shows the multivariate logistic regression analysis of the predictors of 12-month smoking cessation. Indeterminate or suspicious malignant lung nodules on LDCT were associated with a higher 12-month CAR (OR, 3.02; 95% CI, 1.15–7.98). The psychiatric disease was associated with a lower 12-month CAR (OR, 0.06; 95% CI, 0.03–0.15).

## 4. Discussion

In this study, the 12-month CAR of the participants in the residential smoking cessation camp was 31.5%. In baseline LDCT, indeterminate or suspicious malignant lung nodules were associated with a higher 12-month CAR; however, they were not associated with a 6-month CAR. Psychiatric disease in the study participants was associated with lower 6-and 12-month CARs.

Several studies have investigated the effect of CT screening results on smoking cessation; however, they have shown mixed results. Some studies have reported that the quit rate was higher in participants with abnormal findings on LDCT than in those with normal findings, which is in line with our results [14,15,16,20]. A study that used data from the NLST showed that participants with abnormal findings in the LDCT had a higher success rate of smoking cessation than those with normal findings; in particular, more severe findings or changes in LDCT had stronger associations with smoking cessation [14]. In a recent study, Clark et al. reported that false-positive screening results were associated with increased abstinence and less relapse at the 5-year follow-up [15]. Moreover, in the UK Lung Cancer Screening Trial, participants who had abnormal findings that required additional clinical investigation had a higher quit rate than those with negative results [16]. A study from the Danish Lung Cancer Screening trial reported that 1-year quit rates were higher in participants with abnormal CT findings who required a repeat scan 3 months later [20]. In contrast, a study from the NELSON trial showed that the results of LDCT had no statistically significant effect on smoking abstinence in males. However, they found a statistically insignificant increase in the abstinence rate for one or more indeterminate test results compared to only negative test results [17].

In this study, of 30 study participants who had indeterminate or suspicious nodules, 29 participants (96.7%) had indeterminate nodules, and 1 participant (3.3%) had a suspicious nodule. Smoking cessation of participants who have indeterminate nodules could contribute to preventing lung cancer. Furthermore, in lung cancer patients, smoking increases the risk of recurrence and death after cancer treatment [21]. All smokers who undergo lung screening should be encouraged to quit smoking.

Unlike previous lung cancer screening programs that provided brief smoking cessation counseling to participants, we provided smoking cessation counseling with pharmacological treatment for up to 6 months after the completion of residential smoking cessation programs. Therefore, in our study, it was difficult to clearly distinguish the effect of intensive smoking cessation counseling during this period and the effect of smoking cessation due to LDCT findings. In this study, the abnormal LDCT findings (indeterminate or suspicious malignant lung nodules) were not associated with the 6-month CAR but were associated with a higher 12-month CAR.

As with other notable findings of this study, participants with emphysema on LDCT tended to have a lower 12-month CAR. It is possible that the participants did not recognize the harmfulness of emphysema compared to lung nodules [22], and physicians unconsciously more focused on the health risks of cancer to patients compared to those of emphysema. In the recent COVID-19 pandemic, populations with underlying lung diseases are at risk of severe COVID-19 progression and death [4]. Therefore, providing motivation for smoking cessation through a sufficient explanation of abnormal screening findings is important.

In this study, psychiatric history was inversely associated with both the 6- and 12-month CARs. Previous studies have shown that the smoking cessation rate of patients with mental illness is lower than that of the population without mental illness [23,24]. Mental illness is related to various smoking-related risk factors, such as poverty, low education, and unemployment. In addition, the lack of proper recognition of the health effects of smoking, lack of will and confidence, and restrictions on access to smoking cessation programs might be obstacles to quitting smoking among psychiatric patients [25].

In addition, we found that higher education levels and performing regular exercise were associated with higher 6-month smoking cessation rates. Some studies have investigated the association between educational level and smoking status. A nationwide Japanese survey reported that the adjusted prevalence ratio of current smoking was higher in junior high school graduates than in university graduates [26]. Smokers with a higher educational level had a higher rate of smoking cessation [27]. This was because education on the harmful effects of smoking was possible for highly educated people.

Regular exercise may decrease symptoms of cigarette withdrawal and cravings. Some observational studies have reported that physical activity helps with smoking cessation and reduces relapse [28,29]. However, a systematic review, including randomized controlled trials, showed that exercise plus smoking cessation support did not improve long-term abstinence for at least 6 months compared to smoking cessation support alone [30]. A recent randomized controlled trial reported that telephone-delivered exercise counseling with usual care (behavioral counseling and NRT) failed to improve smoking abstinence rates compared to usual care alone; however, they found that more intervention calls successfully delivered were associated with a lower probability of smoking [31]. Further clinical trials are required to clarify whether exercise improves the success rate of smoking cessation.

The strengths of our study are as follows [1]. This is the first study in South Korea to examine the association between lung screening results and smoking cessation. Our study adds to the evidence regarding the association between CT screening results and smoking habits. Further [2], we presented a wide range of information on demographic factors, lifestyle factors, and comorbidities of the study participants. In addition, our results remained significant after adjusting for these covariates. Despite its strengths, this study has some limitations. First, 1-year smoking cessation was determined through telephone interview (self-report) and was not biologically confirmed through examination. Second, we selected participants from smoking cessation camps conducted in one regional tobacco control center, and a relatively small number of subjects and shorter follow-up period is one of the limitations. Third, because we considered unresponsive participants as relapses, there may have been a misclassification bias. Fourth, there was no direct comparison group who did not undergo lung screening in a residential smoking cessation program. In terms of indirect comparison with smokers who visited the Korea National Health Insurance Service smoking cessation program 2016 in primary care clinics, their 6- and 12-month CARs were 39% and 23.4% [32], which were relatively lower quit rates compared to our results.

## 5. Conclusions

The 12-month CAR of participants in the residential smoking cessation program was 31.5%. Indeterminate or suspicious malignant lung nodules were associated with a higher 12-month CAR, whereas a psychiatric history was associated with a lower 12-month CAR. These results suggest that the explanation of abnormal lung screening results for smokers can encourage smoking cessation as a “teachable moment”. Especially in the recent COVID-19 pandemic, smokers and patients with smoking-related diseases are at risk of severe COVID-19 infection. In this regard, smoking cessation counseling is essential in lung screening programs.

## Figures and Tables

**Table 1:** Characteristics of study subjects according to 6 and 12 months of smoking cessation success.

Variables	6 Months		*p*-Value	12 Months		*p*-Value
	Success	Fail		Success	Fail	
	179 (61.3)	113 (38.7)		92 (31.5)	200 (68.5)	
Age (yr, mean ± SD)	55.38 (10.46)	55.02 (11.16)	0.779	53.87 (9.67)	55.87 (11.14)	0.139
Sex			0.113			0.092
Men	169 (94.4)	101 (89.4)		89 (96.7)	181 (90.5)	
Women	10 (5.6)	12 (10.6)		3 (3.3)	19 (9.5)	
Education level			0.022			0.451
Middle school or less	14 (7.8)	21 (18.6)		8 (8.7)	27 (13.5)	
High school	70 (39.1)	38 (33.6)		37 (40.2)	71 (35.5)	
College or beyond	95 (53.1)	54 (47.8)		47 (51.1)	102 (51.0)	
Alcohol consumption			0.777			0.433
Yes	92 (51.4)	60 (53.1)		51 (55.4)	101 (50.5)	
Regular Exercise			0.023			0.571
Yes	69 (38.5)	29 (25.7)		33 (35.9)	65 (32.5)	
Hypertension	52 (29.1)	40 (35.4)	0.255	27 (29.3)	65 (32.5)	0.590
Diabetes Mellitus	32 (17.9)	22 (19.5)	0.733	14 (15.2)	40 (20.0)	0.328
Dyslipidemia	53 (29.6)	20 (17.7)	0.022	29 (31.5)	44 (22.0)	0.081
Coronary artery disease	13 (7.3)	12 (10.6)	0.318	8 (8.7)	17 (8.5)	0.956
Cerebrovascular disease	11 (6.1)	1 (0.9)	0.033	8 (8.7)	4 (2.0)	0.011
Cancer	12 (6.7)	7 (6.2)	0.864	6 (6.5)	13 (6.5)	0.994
Psychiatric disease	37 (20.7)	91 (80.5)	<0.001	7 (7.6)	121 (60.5)	<0.001
Smoking pack-years (mean ± SD)	35.84 (14.19)	40.92 (19.99)	0.020	35.79 (11.54)	38.73 (18.72)	0.102
FTND ^(a)^ score			0.016			0.665
0	3 (1.7)	2 (1.8)		1 (1.1)	4 (2.0)	
Mild [1,2,3]	65 (36.3)	22 (19.5)		26 (28.3)	61 (30.5)	
Moderate [4,5,6]	68 (38.0)	53 (46.9)		43 (46.7)	78 (39.0)	
Severe [7,8,9,10]	43 (24.0)	36 (31.9)		22 (23.9)	57 (28.5)	
Pharmacotherapy			0.275			0.233
No	8 (4.5)	5 (4.4)		1 (1.1)	12 (6.0)	
NRT ^(b)^	67 (37.4)	49 (43.4)		41 (44.6)	75 (37.5)	
Varenicline	6 (3.4)	8 (7.1)		4 (4.3)	10 (5.0)	
Varenicline + short term NRT ^(b)^	98 (54.7)	51 (45.1)		46 (50.0)	103 (51.5)	

Data are presented as the mean ± standard deviation or as the frequency (percentage). ^(a)^ FTND: Fagerstrom Test for Nicotine dependence, ^(b)^ NRT: Nicotine replacement therapy.

**Table 2:** The results of low-dose CT screening of study subjects.

Variables	6 Months		*p*-Value	12 Months		*p*-Value
	Success	Fail		Success	Fail	
	179 (61.3)	113 (38.7)		92 (31.5)	200 (68.5)	
Low-dose computed tomography						
Nodule			0.001			<0.001
Negative	93 (52.0)	63 (55.8)		43 (46.7)	113 (56.5)	
Benign	59 (33.0)	47 (41.6)		27 (29.3)	79 (39.5)	
Indeterminate or suspicious malignancy	27 (15.1)	3 (2.7)		22 (23.9)	8 (4.0)	
Emphysema	49 (27.4)	36 (32.1)	0.384	20 (21.7)	65 (32.7)	0.057
Interstitial lung disease	7 (3.9)	0	0.046	2 (2.2)	5 (2.5)	1.000
Extrapulmonary neoplasm	7 (3.9)	1 (0.9)	0.157	4 (4.3)	4 (2.0)	0.266
Coronary artery calcification	56 (31.3)	22 (19.5)	0.026	20 (21.7)	58 (29.0)	0.193

Data are presented as the frequency (percentage).

**Table 3:** Multivariable logistic regression analysis of predictors for 6 months smoking cessation.

Variables	Unadjusted		Model 1		Model 2	
	OR	95% CI	OR	95% CI	OR	95% CI
Age	1.00	(0.98–1.03)	1.00	(0.9–1.03)	1.03	(1.00–1.06)
Sex						
Men	1		1		1	
Women	0.50	(0.21–1.19)	0.50	(0.21–1.19)	1.67	(0.55–5.16)
Education level						
Middle school or less	1		1		1	
High school	2.76	(1.26–6.05)	3.16	(1.36–7.33)	4.39	(1.36–14.14)
College or beyond	2.64	(1.21–5.61)	3.12	(1.34–7.28)	4.37	(1.36–14.08)
Regular Exercise						
Yes	1.82	(1.08–3.05)	1.79	(1.07–3.02)	2.41	(1.22–4.76)
Dyslipidemia	1.96	(1.10–3.49)	2.01	(1.12–3.61)	1.77	(0.80–3.96)
Cerebrovascular disease	7.33	(0.93–57.60)	6.90	(0.88–54.40)	8.50	(0.75–96.11)
Psychiatric disease	0.06	(0.04–0.11)	0.06	(0.03–0.11)	0.06	(0.03–0.12)
FTND ^(a)^ score	0.98	(0.97–0.99)	0.88	(0.80–0.98)	0.88	(0.77–1.00)
Low-dose computed tomography						
Nodule						
Negative	1		1		1	
Benign	0.85	(0.52–1.40)	0.80	(0.48–1.35)	0.79	(0.40–1.57)
Indeterminate or suspicious malignancy	6.10	(1.77–20.96)	5.67	(1.64–19.59)	1.31	(0.34–5.12)
Coronary artery calcification	1.88	(1.07–3.31)	1.94	(1.08–3.50)	1.91	(0.89–4.12)

Data are presented as odds ratio (95% confidence interval), ^(a)^ FTND: Fagerstrom Test for Nicotine dependence, Model 1: adjusted for age and sex, Model 2 adjusted for age, sex, education level, regular exercise, dyslipidemia, cerebrovascular disease, psychiatric disease, FTND score, lung nodule, coronary artery calcification.

**Table 4:** Multivariable logistic regression analysis of predictors for 12 months smoking cessation.

Variables	Unadjusted		Model 1		Model 2	
	OR	95% CI	OR	95% CI	OR	95% CI
Age	0.98	(0.96–1.01)	0.98	(0.96–1.01)	0.99	(0.96–1.24)
Sex						
Men	1		1		1	
Women	0.32	(0.09–1.11)	0.33	(0.09–1.04)	0.60	(0.14–2.62)
Cerebrovascular disease	4.67	(1.37–15.92)	4.82	(1.40–16.68)	3.24	(0.77–13.63)
Psychiatric disease	0.05	(0.02–0.12)	0.06	(0.02–0.13)	0.06	(0.03–0.15)
FTND ^(a)^ score	0.93	(0.90–1.10)	1.01	(0.91–1.13)	1.09	(0.96–1.24)
Nodule						
Negative	1		1		1	
Benign	0.90	(0.51–1.57)	0.99	(0.55–1.78)	1.03	(0.53–2.01)
Indeterminate or suspicious malignancy	7.23	(2.99–17.46)	7.25	(2.95–17.81)	3.02	(1.15–7.98)
Emphysema	0.57	(0.32–1.02)	0.58	(0.32–1.07)	0.58	(0.28–1.19)

Data are presented as odds ratio (95% confidence interval), ^(a)^ FTND: Fagerstrom Test for Nicotine dependence, Model 1: adjusted for age and sex, Model 2 adjusted for age, sex, cerebrovascular disease, psychiatric disease, FTND score, lung nodule, emphysema.

## Data Availability

The raw data were generated at the Korea Health Promotion Institute. Derived data supporting the findings of this study are available from the corresponding authors on reasonable request.
